# Assessing geographic and institutional disparities in United States diabetic eye disease clinical trials (2000–2023)

**DOI:** 10.1038/s41433-025-03791-5

**Published:** 2025-04-05

**Authors:** Jainam Shah, Sachin Pathuri, Jason Zheng, Ibrahim Furkan Acar, Elanit Safaei, Sailesh Tummala

**Affiliations:** 1 Albert Einstein College of Medicine, Bronx, NY USA; 2 Creighton University School of Medicine, Phoenix, AZ USA; 3 California University of Science and Medicine, Colton, CA USA; 4 University of California, Los Angeles, CA USA; 5 Institute for Society and Genetics, Human Biology and Society, UCLA, Los Angeles, CA USA; 6 Mayo Alix Clinic School of Medicine, Phoenix, AZ USA

**Keywords:** Epidemiology, Retinal diseases, Epidemiology

## Introduction

Disparities in diabetic eye disease clinical trial distribution limit patient representation and equitable access to novel therapeutics. Studies have highlighted geographic maldistributions of clinical trials among rural and low-income populations [1]. Among diabetic eye diseases, diabetic macular edema (DME) and diabetic retinopathy (DR) are leading causes of vision loss [2]. This study evaluates DME and DR clinical trials in the U.S. to identify disparities in geographic and institutional representation.

## Methods

Phase II–IV clinical trials for DME and DR with $\ge$50 participants, initiated between January 1, 2000, and December 31, 2023, were obtained from ClinicalTrials.gov for this cross-sectional study. Multicentre trials were excluded from geographic analysis due to missing site-specific enrolment. Trials were categorized by U.S. Census Bureau region (South, Midwest, West, Northeast) and institution (academic, public, private). Proportions with 95% confidence intervals (CIs) assessed trial distribution, while odds ratios (ORs) and relative risk (RR) compared institutional affiliations between diseases. Multinomial logistic regression evaluated predictors of institutional affiliation. *P*-values assessed significance for categorical comparisons; statistical analysis was conducted in SPSS (version 29, IBM, Chicago, IL). This study protocol was exempt from institutional review board approval by the Albert Einstein College of Medicine, due to use of public, de-identified data, following STROBE guidelines.

## Results

A total of 83 DR and 43 DME trials were included. Among DR trials, 32.5% (95% CI, 22.5%–42.6%) reported geographic sites, compared to 6.9% (2.3%–12.6%) of DME trials (*P*  <  .001). DR trials were mainly private (45.8%, 95% CI: 35.1%–56.5%), followed by public (33.7%, 23.6%–43.9%) and academic institutions (15.7%, 8.1%–23.3%). DME trials were mostly public (58.1%, 43.4%–72.9%), followed by private (34.9%, 21.0%–48.7%) and academic institutions (7.0%, 2.0%–12.0%). The geographic distribution of DR and DME trials (Table 1) was unequal across regions (*P*  <  .001). DR trials were concentrated in the South at 37.0% (18.8%–55.3%) and Midwest at 37.0% (18.8%–55.3%), with fewer in the West (14.8%, 1.4%–28.2%) and Northeast (11.1%, 5.81%–19.34%). DME trials occurred in the South (75.0%, 50.2%–99.8%) and West (25.0%, 5.1%–44.9%), with none in the Midwest or Northeast. DR trials were more likely to be privately sponsored (OR: 4.193, 95% CI: 1.609–10.924) than DME trials (Table 2), and disease type predicted institutional affiliation (*P* = 0.011) on multinomial logistic regression.

**Table 1:** Geographic and Institutional Distribution of DR and DMO U.S. Trials (2000–2023).

Category	Subcategory	DR trials	DR trials (95% CI)	DME trials	DME trials (95% CI)	*P*-value
Geographic distribution	South	37.0%	18.8–55.3%	75.0%	50.2–99.8%	<0.001
	Midwest	37.0%	18.8–55.3%	0%	–	<0.001
	West	14.8%	1.4–28.2%	25.0%	5.1–44.9%	<0.001
	Northeast	11.1%	5.81–19.34%	0%	–	<0.001
Institutional sponsorship	Private	45.8%	35.1–56.5%	34.9%	21.0–48.7%	0.058
	Public	33.7%	23.6–43.9%	58.1%	43.4–72.9%	<0.001
	Academic	15.7%	8.1–23.3%	7.0%	2.0–12.0%	0.19
	Unknown	4.8%	0.2–9.4%	0%	–	0.550

**Table 2:** ORs and RRs for Institutional Affiliations of DR and DMO U.S. Trials (2000–2023).

Disease and category	Subcategory	Odds ratios (ORs)	ORs (95% CI)	Relative Risk (RR)	RRs (95% Cis)	*P-*value
DR institutional sponsorship	Private	4.193	1.609–10.924	2.630	1.319–5.244	0.002
	Public	0.489	0.211–1.132	0.695	0.452–1.069	0.093
	Academic	0.909	0.173–4.765	0.915	0.195–4.293	0.910
DME institutional sponsorship	Private	0.238	0.092–0.622	0.627	0.458–0.859	0.002
	Public	2.04	0.883–4.74	1.423	0.932–2.174	0.093
	Academic	1.10	0.210–5.78	1.006	0.901–1.124	0.910

## Discussion

Excluding multicentre trials limited geographic analysis due to unavailable location-specific data, which may misrepresent geographic diversity when enrolment is disproportionately concentrated at select locations. Previous studies show discrepancies in reporting location-specific data despite data verification, complicating geographic assessment [3]. The predominance of private-sector DR trials (45.8%) and public-sector DME trials (58.1%) implies funding-driven biases. Limited academic involvement in DR (15.7%) and DME trials (7.0%) shows underrepresentation of non-commercial research, consistent with studies showing limited industry-funding to academic centers [4]. DME trials were concentrated in the South, with none in the Northeast or Midwest, reflecting regional disparities in access to emerging therapies. Social determinants of health, such as socioeconomic status and geographic access, may also influence diabetic retinopathy management and contribute to trial access disparities [5]. Policies should promote equitable geographic trial distributions and enhance academic engagement to ensure balanced representation.

## Summary

### What is known about this topic?

- Diabetic retinopathy (DR) and diabetic macular edema (DME) are leading causes of vision loss and are frequently studied in clinical trials.
- Prior research has identified geographic disparities in trial access, particularly among rural and low-income populations.
- The institutional sponsorship landscape of DR and DME trials has been understudied, with little known about the roles of public, private, and academic sectors.

### What this study adds?

- DME trials were disproportionately located in the Southern U.S., with no representation in the Northeast or Midwest, highlighting regional access gaps.
- DR trials were significantly more likely to be privately sponsored, while DME trials were led primarily by public institutions.
- Academic institutions were underrepresented in both DR and DME trials, suggesting potential barriers to noncommercial research participation.

## Data Availability

The data supporting this study’s findings are publicly available from ClinicalTrials.gov. Exclusion criteria were applied to the data obtained from ClinicalTrials.gov for this particular study. The data used for this study’s analysis will be available from the authors upon reasonable request for research purposes if necessary.
